# Hypovitaminosis D among Blood Samples of Patients Presenting to the Department of Biochemistry of a Tertiary Care Center

**DOI:** 10.31729/jnma.8319

**Published:** 2023-11-30

**Authors:** Binaya Tamang, Buddhi Raj Pokhrel, Jharana Shrestha, Narayan Gautam, Binit Kumar Sharma

**Affiliations:** 1 Department of Biochemistry, Universal College of Medical Sciences and Teaching Hospital, Bhairahawa, Rupandehi, Nepal

**Keywords:** *prevalence*, *vitamin D*, *vitamin D deficiency*

## Abstract

**Introduction:**

Hypovitaminosis D is a global public health problem affecting approximately one billion people, with a particularly high prevalence in South Asia. Several hospital-based studies from Nepal show a high prevalence of hypovitaminosis D. However, large-scale community-based studies are lacking. The aim of the study was to find out the prevalence of hypovitaminosis D among blood samples of patients presenting to the Department of Biochemistry of a tertiary care centre.

**Methods:**

A descriptive cross-sectional study was conducted among blood samples of patients presenting to the Department of Biochemistry of a tertiary care centre from 3 November 2022 to 30 April 2023 after obtaining ethical approval from the Institutional Review Committee (Reference number: 136/22). Patients of all age groups and genders who were sent for the evaluation of Vitamin D at the laboratory were included. A convenience sampling technique was used. The point estimate was calculated at a 95% Confidence Interval.

**Results:**

Out of 376 patients, hypovitaminosis was seen in 274 (72.87%) (68.38-77.36, 95% Confidence Interval). Vitamin D insufficiency was present in 252 (91.97%) and vitamin D deficiency was present in 22 (8.03%) participants.

**Conclusions:**

The prevalence of hypovitaminosis D was found to be higher than other studies done in similar settings.

## INTRODUCTION

Vitamin D is either acquired by diet or synthesized from the skin upon exposure to sunlight. While the vitamin D receptor (VDR) is widely expressed, its clinical implications, except for skeletal functions, are poorly understood.^1^ Hypovitaminosis D is a global public health problem affecting approximately one billion people, with a particularly high prevalence in South Asia. This could have several repercussions including musculoskeletal dysfunctions, autoimmune disorders, cardiovascular disorders, and a wide range of pathologic complications, including cancer and infectious diseases.^2,3^

Several hospital-based studies from Nepal show a high prevalence of hypovitaminosis D >70%.^4-6^ Large-scale community-based studies are lacking.

The aim of the study was to find out the prevalence of hypovitaminosis D among blood samples of patients presenting to the Department of Biochemistry of a tertiary care centre.

## METHODS

This descriptive cross-sectional study was conducted in the Department of Biochemistry of Universal College of Medical Sciences and Teaching Hospital, Bhairahawa, Rupandehi, Nepal from 3 November 2022 to 30 April 2023. The ethical approval was taken from the Institutional Review Committee of the same institute (Reference number: UCMS/IRC/136/22). Blood samples of patients of all age groups and genders who were sent for the evaluation of vitamin D (25-OH D) at the laboratory were included. Those patients who were on vitamin D supplementation were excluded from the study. A convenience sampling method was used. The sample size was calculated using the following formula:


$$\begin{array}{ll} \text{n} & ={\text{Z}}^{2}\times \frac{\text{p}\times \text{q}}{{\text{e}}^{\text{2}}}\\ & =1.96^{2}\times \frac{0.50\times 0.50}{0.07^{2}}\\ & =267 \end{array}$$

Where,

n = minimum required sample size
Z = 1.96 at 95% Confidence Interval (CI)
p = prevalence taken as 50% for maximum sample calculation
q = 1-p
e = margin of error, 7%

The calculated sample size was 267. After adding a 10% non-response rate, the calculated sample size was 294. However, a sample size of 376 was taken.

The patients were informed in detail about the study and were ensured of the confidentiality of the data. Informed written consent were obtained from the patients. Consent for children and adolescents was obtained from their parents. All the participants were required to fill out a study proforma that included their socio-demographic parameters. Vitamin D level was estimated from the serum sample of the participants by chemiluminescence assay (MAGLUMI 2000). The Vitamin D status was classified based on the manufacturer's manual which is as follows: Deficient: <10 ng/ml, Insufficient: 10-30 ng/ml, Normal: 30-100 ng/ml.

Data was entered in Microsoft Excel 2016 and analysis was done using IBM SPSS Statistics version 16.0. Point estimate was calculated at a 95% CI.

## RESULTS

Among 376 patients, 274 (72.87%) (68.38-77.36, 95% CI) had hypovitaminosis D. Vitamin D insufficiency was present in 252 (91.97%) and vitamin D deficiency was present in 22 (8.03%) participants. The mean vitamin D level was 24.84±11.52 ng/ml. The mean age of the participants was 45.35±15.96 years. A total of 80 (29.20%) were male and 194 (70.80%) were female (Table 1).

**Table 1:** Gender and age-wise distribution (n= 274).

Variables	Vitamin D category	
	Insufficient n (%)	Deficient n (%)
**Gender**		
Male	76 (27.74)	4 (1.46)
Female	176 (64.23)	18 (6.57)
**Age group (years)**		
≤20	15 (5.47)	1 (0.36)
21-40	91 (33.21)	12 (4.38)
41-60	97 (35.40)	5 (1.82)
>60	49 (17.88)	4 (1.46)

## DISCUSSION

Hypovitaminosis was seen in 274 (72.87%) patients. Another similar study reported a prevalence of vitamin D deficiency in the Kathmandu region to be 73.6% and used a similar assay to our study. The cut-off value for vitamin D deficiency (<30 ng/ml) was similar to our cut-off value for hypovitaminosis.^4^ Another study from Kathmandu which used a similar assay as ours reported a much higher prevalence of hypovitaminosis D (85.6%), where 16% were deficient and 69.6% were insufficient. The mean serum vitamin D level was also much lower (19.69±13.68 ng/ml) than our study. This study also used a different cut-off value for deficiency (<20 ng/ml) and insufficiency (20-29 ng/ml) than ours.^5^

Another study using a similar estimation method but a different analyzer reported 48.7% hypovitaminosis prevalence, which is much lower than our study. The mean serum Vitamin D level was however lower than our study at 51.6±16.0 nmol/L (20.64±6.4 ng/ml). The difference could be attributed to different analyzer and cut-off values (<20 ng/ml insufficient and <12 ng/ml deficient) used for estimation and analysis.^7^ Another study from Western Nepal reported a similar prevalence (70.7%) of hypovitaminosis D. However, the prevalence for deficiency (34.8%) and insufficiency (35.9%) was different from our study. This disparity also can be explained by the difference in the cut-off values used (<20 ng/ml deficiency and 20-30 ng/ml insufficiency). The analytical technique and instrument were not mentioned. The mean serum vitamin D level was 26.5±13.9 ng/ml, which is comparable to our study.^6^

Reports from other parts of South Asia show a much higher prevalence of hypovitaminosis D. A study from North India reported a much higher prevalence 88.9%. The cut-off values for deficiency and insufficiency were different from our study.^8^ A study from Pakistan also revealed a high prevalence of 84.7%.^9^ A systematic review and analysis predicted the overall burden to be 68% in South Asia with the highest burden in Pakistan, followed by Bangladesh, India, Nepal, and Sri Lanka, respectively.^3^ In contrast, a meta-analysis from Africa revealed a prevalence of 58.54% which is much lower than for South-Asian regions.^10^

The overall consensus is that, while hypovitaminosis D is a global burden, it is particularly common in South Asia, including Nepal. Geography, skin pigmentation, cultural practices related to deficient sun exposure, and staple diets lacking vitamin D are suggested to be probable reasons behind such high prevalence in the South Asian regions. The detailed analysis of these factors is still lacking, though.^2,3,11^ Whatever the reasons, this certainly represents a huge challenge.

Certain cautions are required while interpreting the results. First, there is no definitive consensus regarding vitamin D deficiency. Just how much is considered deficient, insufficient, normal, or potentially toxic varies widely across guidelines. For instance, reports from the Institute of Medicine, USA (now National Academy of Medicine), the Endocrine Society, various manufacturing companies etc. have their own optimal values for sufficient vitamin D status and deficient state.^12,13^ This is even more complicated by the fact that there is no unanimous agreement regarding the levels of vitamin D that provide extra-skeletal benefits, standardization protocols of various vitamin D assays, and ethnicity/population-wise reference ranges. These factors will certainly alter the recommended dose and desired reference intervals. Furthermore, the lack of crystal-clear benefits of vitamin D supplementation, and reports from underdeveloped and inconsistent studies make it difficult to reach a concordant conclusion.^14,15^

The scenario from Nepal is even more disquieting due to the lack of nationwide prevalence studies and population-based reference levels. So, the national guidelines regarding the optimal status of vitamin D based on larger community based scientific studies are highly recommended. Therefore the findings of this study should be interpreted within a limited context and can not be generalized to the whole population.

## CONCLUSIONS

The prevalence of hypovitaminosis D among patients visiting the Department of Biochemistry of a tertiary care centre is higher than the other studies from similar settings.
